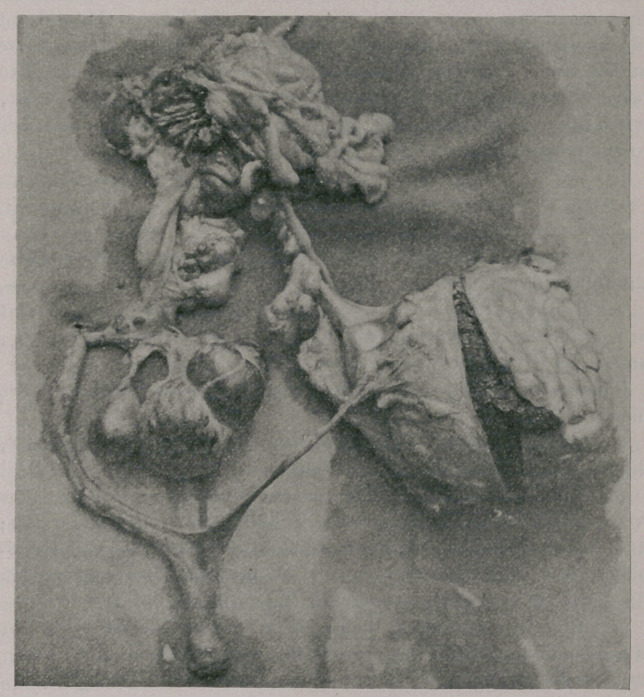# Extra-Uterine Fœtation in the Bitch

**Published:** 1901-11

**Authors:** Cecil French, W. W. Chipman

**Affiliations:** Washington, D. C.; M.D. Edin., Assistant Gynecologist to the Royal Victoria Hospital, Montreal, Canada


### REPORTS OF CASES.

### EXTRA-UTERINE FŒTATION IN THE BITCH.

By Cecil French, D.V.S.,
WASHINGTON, D. C.,
AND
W. W. Chipman, M.D. Edin.,
ASSISTANT GYNECOLOGIST TO THE ROYAL VICTORIA HOSPITAL, MONTREAL, CANADA.

### Notes and History of the Case.

By Cecil French.

During one of my regular visits to the Washington City Pound, in quest of pathological matter from the remains of destroyed dogs, my attention was attracted to a well-bred pointer bitch which had not yet been committed to the lethal chamber. One of the attendants stated that this animal had an internal tumor. On examining her I found a well-nourished body, the external parts being in a healthy condition, with the exception of a cystic formation, about the size of a hen's egg, which was situated over the left hip-joint and contained a sanious matter. Palpation of the abdomen revealed the presence in the cavity of a large, mobile body, firm in consistence and spherical in shape. As there was no history of this case, the animal having been merely brought to the pound to be destroyed, it was out of the question to make a satisfactory diagnosis, but there was no doubt that we were dealing with some form of neoplasm. The animal was committed to the lethal chamber, and immediately afterward I made an examination of the internal organs. On opening the abdominal cavity a very "muddled" condition of the generative organs presented itself. A glance at the accompanying illustration will best convey an idea of the appearance of the parts, but allowance must be made for the broad and round ligaments, which do not figure in the picture. These ligaments on the left side presented a peculiar and wholly unrecognizable appearance, for they were broken up into numerous strands, which were attached at various points on the surface of the spherical body. I removed these strands in order to permit of a better view of the parts, but it was difficult to distinguish them from the much-attenuated left cornu. The course of the left cornu (right in the picture) can be traced up to the surface of the spherical body and apparently beyond it in the direction toward the mass of tissue at the superior extremity of the picture. Associated with this cornu just beyond the spherical body are a number of cysts. The spherical body is in reality a gestation sac. An incision has exposed its interior and contents, the latter consisting of a full-term puppy, one of whose legs projects.

Turning now to the right side of the organs, a few small cysts are observed on the cornu, and suspended, just before the ovary is reached, is a multilocular cyst of considerable dimensions. The ovary of this side is seen, partly exposed by incision, in its sac. Immediately above is the mass of tissue at the superior extremity of the picture. This consists of a portion of the right abdominal wall (where the pin-head appears), a large amount of adventitious tissue, and a small gestation sac, opened by incision to expose a number of small long bones. This sac contained a complete miniature skeleton bathed in a purulent fluid of obnoxious odor, indicating decomposition.

My superficial examination being completed, I photographed the specimen and expressed it to Dr. Adami, of the McGill Pathological Laboratory, who in turn submitted it to Dr. Chipman, whose report is appended below. In the meantime I succeeded in finding the owner of the animal at the time of its death, and obtained from him a partial history, as follows: The bitch was purchased by him at the pound about a year and a half ago. He did not observe the growth for some two weeks, but it was no doubt there at the time. In April of last year he bred her, but she failed to become pregnant. She appeared in heat again last December, when he bred her again, and again she failed. He then took her to the pound, where I found her.

### Pathological Report.

By W. W. Chipman.

The specimen has been removed from the animal, and includes the following:

1. *Body of Uterus.* This body has been divided posteriorly at or near its junction with the vagina, none of the latter being shown; anteriorly it branches into its two cornua.

The uterine body is symmetrical, of good size, and with a patent normal cavity.

2. *Uterine Cornua.* The two cornua are not commensurate, differing both in length and circumference.

(*a*) The right cornu is four inches in length, and its diameter is somewhat disproportionate, as the walls are tumid and thick; its lumen is pervious. The demarcation between cornu and Fallopian tube is not well defined (there is no round ligament on this side, and part of the tube seems to be missing).

Attached by a pedicle to the upper or anterior part of the cornu is a lobulated, multilocular, thin-walled cyst, the contents of which are a uniform light colored serous fluid. Three small single cysts the size of peas are found in the wall of the cornu close at hand. These cysts are the product of degenerate Wolffian remnants. (Bland Sutton has shown that these cysts are not uncommon in domestic animals.)

(*b*) The left cornu is, roughly, double the length of the right and not more than half its circumference.

The diminution in size of the left Müllerian tract begins only anterior to the uterine body (the uterine body is symmetrical, its left half being of equal size to the right), and anteriorly is maintained with marked uniformity only up to the junction of the cornu with its Fallopian tube. (If this defect had been congenital it would probably have implicated the left half of the Müllerian tract in the whole of its extent.)

The junction of the cornu and the Fallopian tube is recognized by the insertion of the round ligament of that side. The cornu is patent throughout its entire length, it is lined by a thin mucosa, and its muscular walls are atrophied.

3. *Fallopian Tubes.* (*a*) The right tube is not intact; neither is the junction between it and its cornu well defined.

(*b*) The left tube is elongated, enlarged, and sacculated. It extends from the insertion of the round ligament of its own side, which occurs at the small gestation sac forward, to its own ovary, which is found adherent to the wall of the large gestation sac. This tube is pervious for the most part, but has been the seat of an old salpingitis. It extends backward to and becomes taken up in the smaller gestation sac, while anteriorly its fimbriated extremity, partly obliterated, is adherent to the large gestation sac.

4. *Ovaries.* (*a*) Right ovary cystic, or, more correctly, fibrocystic, and partly inclosed in its peritoneal capsule.

(*b*) Left ovary healthy, adherent to the wall of the larger gestation sac, and its peritoneal capsule partly taken up in this wall.

5. *Gestation Sacs*—two. (1) Smaller gestation sac: This represents a tubal pregnancy which has occurred in the left Fallopian tube close to the junction of the tube with its own cornu. The walls of this sac are fibrous and thick, and include the distended tubal wall, with a large addition of reactionary fibrous tissue. The gestation sac itself has become adherent, not on its own side of the peritoneal cavity, but on the opposite—the right side. The cavity of the sac is irregular, and the contents a number of bones of an immature fœtus. These bones are partly macerated.

The left cornu leads forward directly to this sac, and continued forward from it is the left Fallopian tube, though neither actually communicates with it.

(2) Larger gestation sac: This gestation sac is for the greater part intraperitoneal, and its wall is largely adventitious, though formed in part from the anterior extremity of the left broad ligament with the left tube and ovary. This sac contains a well-developed full-time female puppy. This puppy, though well preserved, has been dead for some time, as calcareous degeneration has taken place in the placenta and membranes. The membranes themselves cannot be distinguished, and the cord has disappeared. This calcareous deposit is thickest where was once the placental site, but it is continued in the form of thin laminæ completely around the sac. The innermost of these laminæ are stained a rhubarb-yellow with the pigment dissolved from the coat of the yellow-haired puppy.

*Sequence of Events.* The bitch at first was possessed of a normal healthy genital apparatus. Subsequently she suffered from some salpingitis of the left tube. Impregnation occurred, and the fertilized ovum failed to enter the uterine cornu by reason of the effects of the previous salpingitis. It lodged in the left tube and formed therefrom a gestation sac, which became attached to the right side of the peritoneal cavity by inflammatory adhesions. Traction—persistent, steady—was thereby exerted upon the left cornu behind this sac, and this cornu became in consequence attenuated, while the tube in front became sacculated and thick. Gestation went on in this sac until within twenty days or thereby of full time, when, from accident to sac or placenta, the fœtal life was ended. The sac afterward became injected and the contents of the sac reduced to the bones of the fœtus, while the walls became even more fibrous and thick.

Whether or not the larger sac represents a second ovum fertilized at the same insemination it is difficult to say. It may be so, but I rather favor the view that the ovum of the larger sac was fertilized at a later date, probably subsequent to the recovery of the bitch from the death of the first or smaller fœtus, in which case the spermatozoa, going up the right cornu and tube and crossing the peritoneal cavity in the intertubular current, would reach and fertilize an ovum from the left ovary. I favor this view for two reasons:

(*a*) If the two ova had been the yield of the one œstrum, that of the smaller sac having advanced down the tube, we would expect the second ovum also to have advanced at least a certain distance along the tube. There would be at that time nothing to prevent it. But this it does not do.

Subsequently with the formation of the first extra-uterine sac a further salpingitis is set up, and the tube becomes partially occluded, and ova can no longer be carried down its lumen.

Only after the dangers of the first extra-uterine pregnancy are past and the dead fœtus safely encysted does the bitch again permit coitus; and now the spermatozoa, finding their way across from the right tube, meet an ovum lodged at or in the fimbriated ends of the left tube, fertilize it, and an extra-uterine pregnancy, more or less intra-peritoneal, is the result.

(*b*) The death of the smaller fœtus was certainly accompanied by considerable constitutional disturbance to the mother. This disturbance was in all likelihood sufficiently great to terminate the life in the second gestation sac had it then existed. On the contrary, this second larger fœtus came to term, full-sized and well-developed.

*Note.* The fibrocystic ovary on the right side has probably been sterile.

## Figures and Tables

**Figure f1:**